# Impact of DECISION + 2 on patient and physician assessment of shared decision making implementation in the context of antibiotics use for acute respiratory infections

**DOI:** 10.1186/1748-5908-8-144

**Published:** 2013-12-26

**Authors:** France Légaré, Mireille Guerrier, Catherine Nadeau, Caroline Rhéaume, Stéphane Turcotte, Michel Labrecque

**Affiliations:** 1 Research Center of the Centre Hospitalier Universitaire de Québec, Hôpital St-François d’Assise, 10, Rue Espinay, Quebec City, QC G1L 3 L5, Canada; 2 Department of Family Medicine and Emergency Medicine, Université Laval, Quebec City, QC, Canada; 3 Research Center of Institut Universitaire de Cardiologie et de Pneumologie de Québec, Quebec City, QC, Canada

**Keywords:** Shared decision making, Implementation, Theory of planned behavior, Training

## Abstract

**Background:**

DECISION + 2, a training program for physicians, is designed to implement shared decision making (SDM) in the context of antibiotics use for acute respiratory tract infections (ARTIs). We evaluated the impact of DECISION + 2 on SDM implementation as assessed by patients and physicians, and on physicians’ intention to engage in SDM.

**Methods:**

From 2010 to 2011, a multi-center, two-arm, parallel randomized clustered trial appraised the effects of DECISION + 2 on the decision to use antibiotics for patients consulting for ARTIs. We randomized 12 family practice teaching units (FPTUs) to either DECISION + 2 or usual care. After the consultation, both physicians and patients independently completed questionnaires based on the D-Option scale regarding SDM behaviors during the consultation. Patients also answered items assessing the role they assumed during the consultation (active/collaborative/passive). Before and after the intervention, physicians completed a questionnaire based on the Theory of Planned Behavior to measure their intention to engage in SDM. To account for the cluster design, we used generalized estimating equations and generalized linear mixed models to assess the impact of DECISION + 2 on the outcomes of interest.

**Results:**

A total of 270 physicians (66% women) participated in the study. After DECISION + 2, patients’ D-Option scores were 80.1 ± 1.1 out of 100 in the intervention group and 74.9 ± 1.1 in the control group (p = 0.001). Physicians’ D-Option scores were 79.7 ± 1.8 in the intervention group and 76.3 ± 1.9 in the control group (p = 0.2). However, subgroup analyses showed that teacher physicians D-Option scores were 79.7 ± 1.5 and 73.0 ± 1.4 respectively (p = 0.001). More patients reported assuming an active or collaborative role in the intervention group (67.1%), than in the control group (49.2%) (p = 0.04). There was a significant relation between patients’ and physicians’ D-Option scores (p < 0.01) and also between patient-reported assumed roles and both D-Option scores (as assessed by patients, p < 0.01; and physicians, p = 0.01). DECISION + 2 had no impact on the intention of physicians to engage in SDM.

**Conclusion:**

DECISION + 2 positively influenced SDM behaviors as assessed by patients and teacher physicians. Physicians’ intention to engage in SDM was not affected by DECISION + 2.

**Trial registration:**

ClinicalTrials.gov trials register no. NCT01116076.

## Background

Despite recent efforts to decrease prescription rates, antibiotics are still too frequently prescribed for acute respiratory tract infections (ARTIs) [1]. Results of attempts to improve the clinical decision making process regarding the use of antibiotics for ARTIs have been weakly conclusive, and interventions to reduce the use of antibiotics have mostly shown only modest improvements [2,3]. Scientific uncertainty about use of antibiotics as well as a failure to take into account the perspectives of both parties (patient as well as health professional), each with their own kind of expertise, may explain these results. In this clinical context, shared decision making (SDM) is an interesting pathway in the pursuit of optimal decisions. DECISION + 2, a training program for physicians, is designed to implement SDM in the context of antibiotics use for ARTIs [4,5].

Engaging in SDM consists of encouraging patients to participate in the decision making process while sharing and reviewing patient values and preferences and the relative importance of the benefits and risks associated with treatment options. SDM encompasses a series of steps, each of which can be referred to as a specific SDM behavior [6,7]. In the clinical decision making process, the patient may choose to assume a number of roles, ranging from fully autonomous (patient selects his/her own treatment alone) through truly collaborative (physician and patient share the decision) to passive (physician makes the decision alone or hardly takes the patient’s view into account) [8]. The role the patient assumes that best matches the SDM paradigm is the collaborative role, though moving patients from a passive to a more active role may also be considered an important step toward SDM [9].

Few authors have studied the implementation of SDM in routine clinical settings, and the literature is not yet specific about the most effective types of intervention for increasing healthcare professionals’ adoption of SDM behaviors [10]. While behavior change interventions are essential to improving the practice of clinical medicine, a thorough understanding of the mechanisms underlying SDM behaviors is necessary in order to implement such changes [11,12]. In response to the disappointing results of many implementation interventions, researchers are increasingly using socio-cognitive theories to increase our understanding of underlying behavior mechanisms that might inform the design of further interventions [13]. The Theory of Planned Behavior (TPB) is one of the most frequently used theories for probing the factors that influence any individual behaviors, including those of patients and physicians [14-17]. It states that a specific behavior is mainly explained by the intention to perform it, and the intention itself can be predicted by measuring its three main determinants: attitude, subjective norm and perceived behavioral control [18]. In order to better understand the main results of this trial and better inform future SDM implementation studies, we deemed it essential to assess and reflect on the theoretical underpinnings of the DECISION + 2 intervention. Consequently, as a secondary objective embedded within the main cluster randomized trial (cRT), we used the TPB to evaluate the impact of DECISION + 2 on SDM implementation as assessed by patients and physicians, and on physicians’ intention to engage in SDM.

## Methods

### Study design

The study consisted of a multi-center two-arm parallel CRT in three stages: the baseline data collection (physician and patient recruitment); the intervention (DECISION + 2); and post-intervention data collection (patient recruitment). We conducted the trial in a network of 12 family practices teaching units (FPTUs) in the Department of Family Medicine and Emergency Medicine at Université Laval, Quebec, Canada. The 12 FPTUs were randomly allocated to either an intervention group exposed to DECISION + 2 or a control group with no intervention (usual care) [4]. The randomization was performed by a biostatistician using web-based software. Due to the nature of the trial, blinding was not possible.

### Participants and recruitment procedure

All 12 FPTUs in the network of the Department of Family and Emergency Medicine at Université Laval were eligible to participate. All teacher family physicians, residents and nurse practitioners who provide care in the FPTU walk-in clinics were also eligible. Exclusion criteria included previous participation in the DECISION + pilot project and leave of absence during the study duration. We included patients (adults or children accompanied by a parent or legal guardian) with a diagnosis of acute respiratory infection (bronchitis, otitis media, pharyngitis or rhinosinusitis) and for whom the use of antibiotics was subsequently considered either by the patient or the physician during the visit [5]. Patients in the baseline data collection were different from those in the post-intervention data collection; however the physicians were the same in both data collection periods. All participants signed an informed consent form approved by the review boards of the two health and social services centres involved, namely the Centre de santé et de Services Sociaux de la Vieille-Capitale and the Centre de Santé et de Services Sociaux du Nord de Lanaudière.

### Intervention group

The DECISION + 2 training program includes a web-based self-tutorial, a face-to-face, interactive session using videos, exercises and decision support tools, and a reminder at the point of care. The program was adapted from the original DECISION + pilot training program for the purpose of the present study [19,20]. The web-based self-tutorial lasts about 120 minutes and was intended to develop knowledge and skills regarding the clinical decision making process concerning antibiotic treatments for ARTIs in primary care, including knowledge of: the probabilistic nature of a diagnosis of a bacterial versus a viral infection; scientific evidence regarding the risk/benefit ratios of the options; communication techniques; and strategies to foster patients’ participation in the decision making process [21]. Participants in face-to-face small group interactive sessions reviewed the key components of the web-based self-tutorial to enhance their ability to integrate the process of SDM into their practice regarding the use of antibiotics for ARTIs. Face-to-face small group interactive sessions were given by a principal investigator of the study or by a teaching physician from FPTUs who had been trained beforehand.

### Control group

Physicians and patients in the control group were not exposed to DECISION + 2. Physicians were instructed to provide usual care to eligible patients.

### Data collection and variables

**Figure 1:**
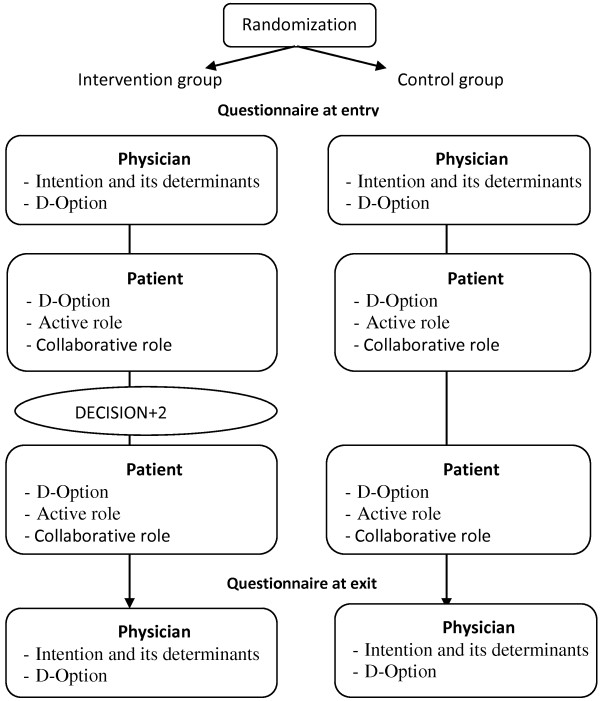
The questionnaire flowchart showing the outcomes assessed at each point of time.

All participants completed self-administered questionnaires before and after DECISION + 2 between July 2010 and April 2011. The questionnaire flowchart is presented in Figure 1. We assessed SDM behaviors after the clinical encounter using the D-Option scale [22,23] and a modified version of the Control Preferences Scale (CPS), which we referred to as ‘assumed role’ [8]. Using a TPB-based scale, we collected data from participating physicians’ intentions to engage in SDM and its related determinants (perceived behavioral control, subjective norm and attitude) at study entry and exit.

The D-Option is a scale used to assess perceived involvement in decision making from the perspective of both the patient and the physician (as opposed to that of a third observer) and includes 10 items. Both patients and their physicians independently completed the D-Option questionnaire after the consultation. We used mean scores out of a total of 100. The modified CPS measures the patient’s assumed role in decision making on a 1 to 5 response scale: 1) ‘I made the decision’; 2) ‘I made the decision after seriously considering my physician’s opinion’; 3) ‘My physician and I shared the responsibility for the decision making’; 4) ‘My physician made the decision after seriously considering my opinion’; 5) ‘My physician made the decision.’ The patient playing an active role is indicated by responses 1 and 2, a collaborative role by 3, and a passive role by 4 and 5. For this study, we coded a new binary variable to combine the collaborative role and the active role (assumed to be SDM behaviors) as distinct from the passive role. Based on the TPB, we assessed physicians’ intention to engage in SDM in the future and its four related determinants: instrumental attitude and affective attitude were assessed by means of three items each, subjective norm by three items, and perceived behavioral control by three items. We used a 7-point Likert scale from -3 (very low) to +3 (very high) to measure the intention and its related determinants. The reliabilities of the scales for behavioral intention and its determinants (instrumental attitude, affective attitude, subjective norm, and perceived behavioral control) were from 0.67 to 0.93. We also collected sociodemographic characteristics of all participating physicians and patients.

### Statistical analysis

We used descriptive statistics to describe characteristics of FPTUs, physicians, their scores of intention (and its determinants) and SDM behaviors. To take the non-independence in the data (clustering effect) into account, we assessed the impact of the intervention on intention (and its determinants) using generalized linear mixed models (GLMMs) and assessed its impact on SDM behaviors using GLMMs for the D-Option scales and generalized estimating equations (GEE) for the patient’s assumed role. We also used GEE and GLMMs to assess the relation among the three SDM behavior measures and to assess the relation between SDM behaviors and physician intention. We performed statistical analysis using the SAS version 9.3 (SAS Institute Inc., Cary, N.C., USA).

## Results

### Flow of the trial and characteristics of participants

**Figure 2:**
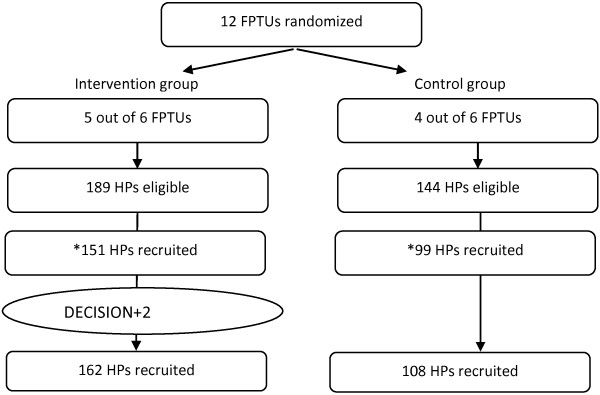
**The flow study of participants showing the number of eligible health professionals in each group.** *We included only the participating physicians who had completed their entry questionnaire before DECISION+2 was delivered. Thus we included 151 physicians in the intervention group and 99 in the control group.

Figure 2 depicts the study flow. Out of the 12 eligible FPTUs, nine participated in the study: four in the control group and five in the intervention group. A total of 108 (68% women) physicians and 162 (63% women) physicians completed the questionnaire in the control and intervention groups respectively. However, we included only the participating physicians who had completed their entry questionnaire before DECISION + 2 was delivered. Thus we included 151 physicians in the intervention group and 99 in the control group (see Figure 2). Participants’ sociodemographic characteristics are reported in Table 1.

**Table 1:** **Characteristics of participating FPTUs**^**1 **^**and physicians according to study groups**

	**n/N (%) of physicians**^**2**^	
	**Control**	**Intervention**
FPTUs^1^	(n = 4)	(n = 5)
Physicians	(n = 108)	(n = 162)
Participating teachers		
Female	36/53 (68)	49/78 (63)
Age, year, mean ± SD	43.7 ± 10	42.0 ± 9.4
Number of years in practice, mean ± SD	15.2 ± 10.7	13.9 ± 10.3
Residents		
Female	34/55 (62)	60/84 (72)
Age, year, mean ± SD	27.3 ± 4.1	27.9 ± 4.5

^1^FPTUs = Family practice teaching units.

^2^Unless otherwise indicated.

### Impact of DECISION + 2 on SDM behaviors as assessed by patients and physicians

At study entry, SDM behaviors as reported by patients and physicians showed similar results (Table 2). At study exit, we observed higher scores of SDM behavior as assessed by both physicians and patients using the D-Option in the intervention group than in the control group, but the difference was only significant for patients (Table 2). Significantly more patients reported assuming an active and collaborative role in the intervention group than in the control group (Table 2). Also, we observed that DECISION + 2 seems to have a greater impact on patients reporting that they assumed an ‘active’ role than a ‘collaborative’ role (data not shown).

**Table 2:** Shared decision making behaviors at entry and exit in the study according to study groups

**Behaviors**	**Entry**		**Exit**		***p*-value**
	**Control**	**Intervention**	**Control**	**Intervention**	
D-Option (Patient)	80.0 ± 1.5	79.3 ± 1.4	74.9 ± 1.1	80.1 ± 1.1	0.001^1^
Mean ± SD					
D-Option (Physician)	75.5 ± 1.7	74.4 ± 2.1	76.3 ± 1.9	79.7 ± 1.8	0.20
Mean ± SD					
Assumed role (patient)					0.04^2^
Active/collaborative role n (%)	99 (57.9)	101 (55.5)	87 (49.2)	118 (67.1)	
Passive role n (%)	72 (42.1)	81 (44.5)	90 (50.8)	58 (32.9)	

^1^Difference between groups at study exit evaluated with generalized linear mixed models to adjust for cluster design.

^2^Difference between groups at study exit evaluated with generalized estimating equations to adjust for cluster design.

Subgroup analysis on family medicine residents and teacher physician groups revealed that patients reported a higher impact of DECISION + 2 on SDM behavior assessed using D-Option with teacher physicians (intervention group: 79.7 ± 1.5 vs. control group: 73.0 ± 1.4; p = 0.001) than with residents (intervention group: 79.9 ± 1.4 vs. control group: 77.4 ± 1.5; p = 0.21).We observed no other statistically different impact in the subgroup analysis, and the results were similar to those for all the participants (*i.e*., residents and teacher physicians).

### Impact of DECISION + 2 on the intention to engage in SDM in the future

Results of intention and its related determinants before and after the intervention are shown in Table 3. The mean scores of all TPB-based determinants of intention were high (there were no negative scores). We found no significant difference in mean scores of the determinants of intention between the control group and the intervention group after DECISION + 2.

**Table 3:** Intention to engage in shared decision making and its related determinants at study entry and exit according to study groups

**TPB constructs**	**Entry**		**Exit**		**Mean difference**^**1**^	***p*-value**^**1**^
	**Control**	**Intervention**	**Control**	**Intervention**		
	**Mean ± SD**	**Mean ± SD**	**Mean ± SD**	**Mean ± SD**		
Intention	1.5 ± 0.1	1.6 ± 0.1	1.8 ± 0.1	1.7 ± 0.1	0.1	0.74
Instrumental attitude	1.9 ± 0.1	1.9 ± 0.1	2.2 ± 0.1	2.2 ± 0.1	0	0.97
Affective attitude	1.1 ± 0.2	1.3 ± 0.1	1.4 ± 0.1	1.6 ± 0.1	0.2	0.19
Subjective norm	1.4 ± 0.1	1.5 ± 0.1	1.7 ± 0.1	1.6 ± 0.1	0.1	0.55
Perceived behavioral control	1.1 ± 0.1	1.2 ± 0.1	1.3 ± 0.1	1.3 ± 0.1	0	0.99

^1^Difference between groups at study exit evaluated with generalized linear mixed models to adjust for cluster design.

### Relation between SDM behaviors

Table 4 details the relation between each possible pair of SDM behaviors. There was a statistically significant association between the SDM behaviors as assessed separately by patients and physicians using the D-Option scale (regression coefficient = 0.2, p <0.01). Furthermore, we observed a statistically significant association between the two D-Option scores and the patient-reported assumed role (Physician D-Option: regression coefficient = 3.4, p = 0.01; Patient D-Option: regression coefficient = 6.8, p < 0.01).

**Table 4:** Relation between shared decision making behaviors

**Shared decision making behaviors**				
	**D-Option (patient)**		**D-Option (Physician)**	
	**Regression coefficient ± standard error**	***p*-value**	**Regression coefficient ± standard error**	***p*-value**
D-Option (physician)	0.2 ± 0.1^1^	< 0.01^2^	-	-
Assumed role (patient) (Active or collaborative vs. passive role)	6.8 ± 1.4^3^	< 0.01^4^	3.4 ± 1.2^3^	0.01^4^

^1^Increase in the average value of D-Option (Patient) when the variable D-Option (Physician) increased by one point (out of 100).

^2^Evaluated with generalized linear mixed models to adjust for cluster design.

^3^Increase in the average value of D-Option (out of 100) for a patient who reported an active or collaborative role compared to a patient who reported a passive role.

^4^Evaluated with generalized estimating equations to adjust for cluster design.

### Relation between behavioral intention and SDM behaviors

Table 5 details the relation between physicians’ intention to engage in SDM and each of the measures of SDM behaviors. We observed statistically significant association between physicians’ perceived involvement in SDM, or D-Option (physician) and their intention to engage in SDM at study entry (regression coefficient = 3.5, p < 0.01) and at exit (regression coefficient = 4.3, p < 0.01). However, there was no association between physicians’ intention and their SDM behaviors as assessed by patients, whether using D-Option (patient) or the modified CPS. Subgroup analysis for residents and teacher physicians did not differ statistically between residents and teacher physicians and were similar to the results obtained for both groups combined.

**Table 5:** Relation between shared decision making behaviors and physician intention

**Shared decision making behaviors**						
	**D-Option (patient)**		**D-Option (physician)**		**Assumed role (patient) (Active or collaborative vs. passive role)**	
**Intention (physician)**	**Regression coefficient ± standard error**	***p*-value**	**Regression coefficient ± standard error**	***p*-value**	**Regression coefficient ± standard error**	***p*-value**
Entry	-0.1 ± 0.9^1^	0.89^2^	3.5 ± 0.7^1^	< 0.01^2^	0.0 ± 0.1^3^	0.81^4^
Exit	0.5 ± 1.0^1^	0.60^2^	4.3 ± 0.8^1^	< 0.01^2^	0.1 ± 0.2^3^	0.60^4^

^1^Increase in the average value of D-Option when the variable intention (physician) increased by one point (on a scale of -3 to +3).

^2^Evaluated with generalized linear mixed models to adjust for cluster design.

^3^With every increase of one point on the intention scale, the chances of a patient being active or collaborative rather than passive increased by e ^regression coefficient^ (0% at entry and 11% at exit).

^4^Evaluated with generalized estimating equations to adjust for cluster design.

## Discussion

Our study demonstrated a favorable impact of DECISION + 2 on SDM implementation in clinical practice as assessed by patients and teacher physicians using three measures. To the best of our knowledge, this full cRT is among the first to assess an SDM implementation intervention using the TPB. For example, none of the five trials that were reviewed for the Cochrane Collaboration included the use of the TPB in their trial [10]. In our study, three measures showed a statistically significant positive difference: patient-perceived involvement in SDM regarding the use of antibiotics for ARTIs as measured by D-Option (patient) and D-Option (teacher physician) and patient-reported assumed role in decision making as measured by the modified CPS. There was also a significant association between patient-perceived involvement in SDM and physician-perceived involvement (both D-Option measures), as well as a significant association between patient-reported assumed role (modified CPS) and both patient- and physician-perceived involvement in SDM (using D-Option measures). However, our findings indicate that DECISION + 2 had no impact on the intention of physicians to engage in SDM in the future, even though this intention was slightly associated with SDM behavior as assessed by the physician, or D-Option (physician). The results of a secondary analysis of the DECISION + 2 study has led us to reflect further on the theoretical underpinnings of the DECISION + 2 intervention, and this has increased our understanding of trial results published previously [5]. We therefore suggest that in all future SDM implementation studies, the theoretical underpinnings of the intervention implementation should be described and analyzed for better understanding of results. These results lead us to make four main observations.

First, our results show that it is possible to train physicians to encourage patients to assume a more active role in decision making about their care. In recent years, many healthcare systems have encouraged and empowered patients to be more involved in decision making [24,25]. These findings are an important achievement since many studies have reported that patient involvement in their care has a positive relationship to health status outcomes [26-29]. Our findings suggest that DECISION + 2 has the potential to provide physicians with the skills and competencies needed for SDM to occur during consultation, and that both patients and physicians are responsive to the translation of these newly acquired skills and competencies into clinical practice.

Second, it is interesting to note that patients’ and physicians’ D-Option scores were slightly associated. Although there might be differences in what each party (patient/physician) perceives as SDM behaviors, this association suggests that we should not dismiss tools that measure SDM behaviors from the perspective of both patients and physicians. Our findings suggest that the D-Option assessments (by patients and physicians separately) might provide a valuable measure of SDM behaviors. Our study also shows that SDM behaviors as measured using D-Option from each perspective (patient and physician) are associated with the role assumed by patients as measured using the modified version of the CPS. This provides valuable evidence of the ‘measurement validity’ of both D-Option and assumed roles as measured by the modified CPS. Interestingly, subgroup analysis showed that D-Option assessment by patients revealed that DECISION + 2 had a significant impact on teacher physicians, as observed for the combined groups, but not on the residents, meaning that the impact of DECISION + 2 on the combined groups was greatly influenced by its impact on the teacher physician subgroup. While this result can be interpreted in several different ways, the most likely explanation is that D-Option scores in the resident subgroup were higher than D-Option scores in the teacher physician subgroup. This difference with the control group may have been caused by the fact that residents are more aware of new practice methods such as SDM and are also more likely to be sensitive to the on-going evaluation they are experiencing when training in the FPTUs.

Third, in considering descriptive statistics of patient-reported assumed role (data not shown), we observed that DECISION + 2 seems to have a greater impact on patients reporting that they assumed an ‘active’ role than a ‘collaborative’ role. This difference may reflect a difference, from the patients’ point of view, between being involved in the decision making and sharing it equally. Authors of an earlier study have pointed out that patients seem to distinguish between the process of involvement and who makes the final decision [30]. Another study found that patients who opted against cancer screening after reviewing a brochure or decision aid were less likely to discuss their decision with their physician; *i.e*., from the patients’ point of view, they may be active in the decision without requiring any participation by the physician at all [31]. Therefore, it may be useful in future SDM implementation work to better distinguish between measuring the extent to which the patient engages in decision making (or moves towards a more active role in decision making) and the occurrence of SDM (where patient and physician make the decision together). As mentioned earlier, the ‘active role’ appears better aligned with an informed decision than a shared decision. Shared decision making refers to the middle ground between an informed decision making process and a paternalistic decision making process [32]. As demonstrated by the extensive use of the Control Preference Scale and Assumed Role in SDM studies [33], favouring an SDM process may lead to setting an optimal level of autonomy in patients so that they are never at the extreme end of the continuum: *i.e.*, they never report having made the decision alone. Future research on the conceptualization of SDM should focus on this.

Fourth, we did not see any impact of DECISION + 2 on the intention of physicians to engage in SDM in the future or on its determinants as defined by the TPB. The following points may provide an explanation. First, there was a ceiling effect, with physicians showing high scores on all these measures before the intervention. Since patient and public involvement is getting popular and physicians are more aware of SDM, it is not surprising that we observed high intention, as other studies have done [17,34]. Second, the TPB defines and distinguishes between several precise behaviors in great detail [35]. In our study, SDM behaviors were defined broadly, while engaging in SDM in fact encompasses many competencies, or more specifically a set of SDM behaviors. On the other hand, SDM behaviors as assessed by physicians after consultations with single patients were shown to be associated with their intention to engage in SDM at study entry and exit, thus providing some grounds for anticipating that this link between intention and behaviour will continue to be trustworthy in the future [14-17]. Lastly, in the case of highly recommended or desirable behaviors with high ceiling effects, the TPB may not be adequate for understanding behavior change.

This study has a number of limitations. First, it was embedded in a larger study and was not designed nor powered for our stated objective. Second, our results regarding patient involvement in decision making could have been overestimated due to the use of patient and physician self-reported measures, a problem that could be overcome by using an objective measurement such as third-observer instrument (*e.g*., the OPTION observer instrument). Third, physicians practicing in FPTUs are not representative of the whole population of physicians and may be more aware of SDM since they are immersed in an academic environment where SDM and patient involvement is valued throughout the curriculum. Physicians who participated may be more interested in SDM, which may have led to a weak overestimation of the impact of DECISION + 2 on the entire physician population (ceiling effect). However, due to the high rate of participation, the impact should be negligible. We did not expect much contamination between groups because physicians needed a personal access code to the web-based tutorial and we also made sure to take attendance during the live workshop. Finally, as discussed above, we can presume that a desirability bias occurred because of the increasing popularity of and public pressure for patient and public involvement programs, as suggested by the high scores of intention and its determinants such as attitude [34,36].

## Conclusions

Our findings suggest that although engaging in SDM may be a complex behavior for physicians to adopt, DECISION + 2, a training program, has the potential to provide physicians with the skills and competencies needed for the successful implementation of SDM in clinical practice. The results also indicate that both patients and physicians are responsive to the translation of these newly acquired skills and competencies into their behaviour during consultations, and that both parties share some common understanding of SDM behaviors.

## Abbreviations

ARTIs: Acute respiratory treat infections; CPS: Control preference scale; cRT: Cluster randomized trial; FPTUs: Family practice teaching units; SDM: Shared decision making; TPB: Theory of planned behavior.

## Competing interests

The authors declare that they have no competing interests.

## Authors’ contributions

All authors made substantial contributions to the interpretation of the data. ML and FL planned and coordinated the study. FL, MG and CN wrote the first draft of the manuscript. ST and MG analyzed the data and performed statistical analyses. All authors reviewed and approved the final manuscript.
